# The Link Between Dysbiosis, Inflammation, Oxidative Stress, and Asthma—The Role of Probiotics, Prebiotics, and Antioxidants

**DOI:** 10.3390/nu17010016

**Published:** 2024-12-24

**Authors:** Paulina Kleniewska, Rafał Pawliczak

**Affiliations:** Department of Immunopathology, Faculty of Medicine, Medical University of Lodz, Zeligowskiego 7/9, 90-752 Lodz, Poland; rafal.pawliczak@csk.umed.lodz.pl

**Keywords:** oxidative stress, antioxidants, probiotics, prebiotics, asthma

## Abstract

**Background:** Asthma (a chronic inflammatory disease of the airways) is characterized by a variable course, response to treatment, and prognosis. Its incidence has increased significantly in recent decades. Unfortunately, modern lifestyle and environmental factors contribute to the further increase in the incidence of this disease. Progressive industrialization and urbanization, widespread use of antibiotic therapy, excessive sterility and inappropriate, highly processed diets are some of the many risk factors that are relevant today. Over the years, a lot of evidence has been gathered showing the influence of microorganisms of the gut or airways on human health. Studies published in recent years indicate that dysbiosis (microbial imbalance) and oxidative stress (pro-oxidant–antioxidant imbalance) are important elements of the pathogenesis of this inflammatory disease. Scientists have attempted to counteract the effects of this process by using probiotics, prebiotics, and antioxidants. The use of probiotic microorganisms positively modulates the immune system by maintaining homeostasis between individual fractions of immune system cells. Moreover, recently conducted experiments have shown that probiotics have antioxidant, anti-inflammatory, and protective properties in oxidative stress (OS). The aim of this study is to present the current state of knowledge on the role of dysbiosis and OS in the pathogenesis of asthma. **Conclusions:** This review highlights the importance of using probiotics, prebiotics, and antioxidants as potential strategies to support the treatment and prevention of this disease.

## 1. Introduction

Asthma is one of the most common chronic respiratory diseases [1] that affects all age groups. One in five child and adolescent asthma patients and one in eight adult asthma patients are estimated to experience uncontrolled symptoms that reduce their quality of life [2]. The disease cannot be completely cured, so treatment focuses primarily on alleviating symptoms using inhaled glucocorticosteroids, long-acting or short-acting beta-2-agonists [3,4,5]. Biological treatment [6,7,8], including monoclonal antibodies, has only been used to a limited extent.

In recent years, the number of studies focusing on the role of microorganisms in health and in the pathogenesis of diseases, including asthma, has increased rapidly. In 2016, scientists reported that increased amounts of *Lachnospira* and *Clostridium neonatale* are associated with childhood asthma. Moreover, in children about one year of age with recurrent wheezing, higher levels of *C. difficile*-specific IgG were found [9]. Recent reports showed a higher number of these bacteria in infants with recurrent wheezing during the first two years of life [10]. Fujimura et al. [11] have reported that newborns with lower relative abundance of *Bifidobacteria*, *Akkermansia*, and *Faecalibacterium* and lower relative abundance of Candida and Rhodotorula fungi had a higher risk of developing this disease. Another work describes that only *Faecalibacterium*, *Lachnospira*, *Veillonella*, and *Rothia* are crucial for the subsequent development of asthma [12].

Recently, researchers have also pointed out the involvement of reactive oxygen species in the pathogenesis of asthma. Their high concentrations activate the expression of proinflammatory genes, which leads to the increase in adhesion molecules and the secretion of proinflammatory cytokines and chemokines [13,14].

**Figure 1:**
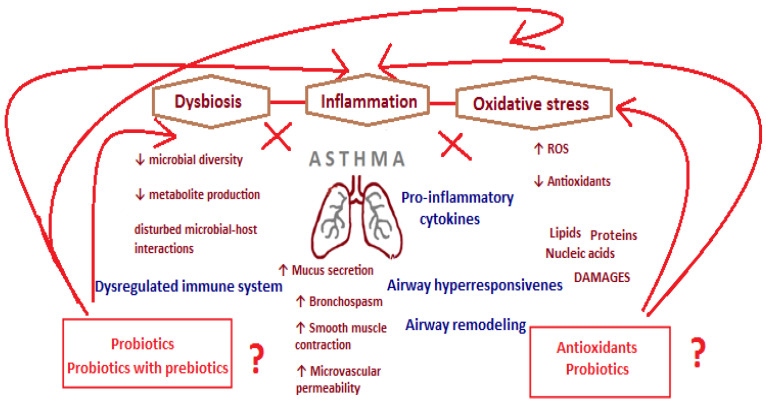
Pathomechanism of changes occurring in the course of asthma. Proposed role of probiotics, prebiotics, and antioxidants in disease treatment and prevention (↑—increase; ↓ decrease/reduction; → influence on).

Based on the published studies, it can be concluded that there is a potential link between inflammation, dysbiosis, and oxidative stress in asthma (Figure 1).

The aim of this study is to present the state of knowledge on the participation of dysbiosis and reactive oxygen species in the development of asthma. Moreover, this paper reviews the latest data on the role of probiotics, prebiotics, and antioxidants in this area.

### 1.1. Asthma as a Health Problem Affecting All Age Groups

Clinically, asthma is characterized by paroxysmal shortness of breath, coughing, chest tightness, and wheezing [3]. Variable and reversible airflow limitation occurs spontaneously and under the influence of physical, chemical, and biological aspects. A characteristic feature of symptomatic asthma is bronchial hyperresponsiveness (BHR) [15]. Importantly, according to the Global Initiative for Asthma (GINA) guidelines, airway hyperresponsiveness (AHR) and inflammation of the airways are not necessary and sufficient to make the diagnosis [3]. Factors contributing to the development of the disease include the following: environmental factors, e.g., allergens (house dust mites, animal allergens, mold and yeast-like fungi, cockroach allergens, pollen of plants, grasses, and trees), tobacco smoke (active and passive smoking), past viral respiratory infections, air pollution (exposure to NO_2_), occupational factors (work environment, exposure to harmful factors), diet, and genetic factors such as atopy or bronchial hyperresponsiveness [3,16].

The classic division of the disease into allergic and non-allergic phenotypes [17] is noteworthy, although it has now been replaced by a classification based on the degree of symptom control. Eosinophils are the main component of the inflammatory infiltrate in the airways. They are responsible for, among others, epithelial damage, bronchial remodeling, and BHR. Importantly, eosinophils are also a source of inflammatory mediators. Cytokines produced by eosinophils include, e.g., interleukins (IL-) 1α, 3, 5, 6, 8; and TNF-α, or TGF-β, which enhance inflammatory processes. Moreover, increased expression of cytokines such IL-4 and IL-13 was found in the bronchial mucosa, sputum, bronchoalveolar lavage fluid (BALF), and peripheral blood of asthmatic patients [18]. Other mediators of the inflammatory reaction, chemokines, growth factors, proteases, and other elements of the innate immune system, tissue regeneration, and repair system are also highly expressed in the bronchial mucosa of asthmatic patients.

The classical theory of the pathophysiology of allergic asthma assumes that it is a disease with Th2-dependent inflammation, i.e., the basis of which is a disturbed balance between the activity of Th1 and Th2 lymphocytes, in favor of the latter. Thus, Th2 lymphocytes initiate and promote the development of bronchial asthma, among others, by secreting proinflammatory interleukins, such as IL-4, -5, -9, and -13. The results of studies conducted in recent years indicate an important role in the etiopathogenesis of allergic asthma of regulatory cells (Tregs), IL-10, IL-17, and TGF-β.

**Figure 2:**
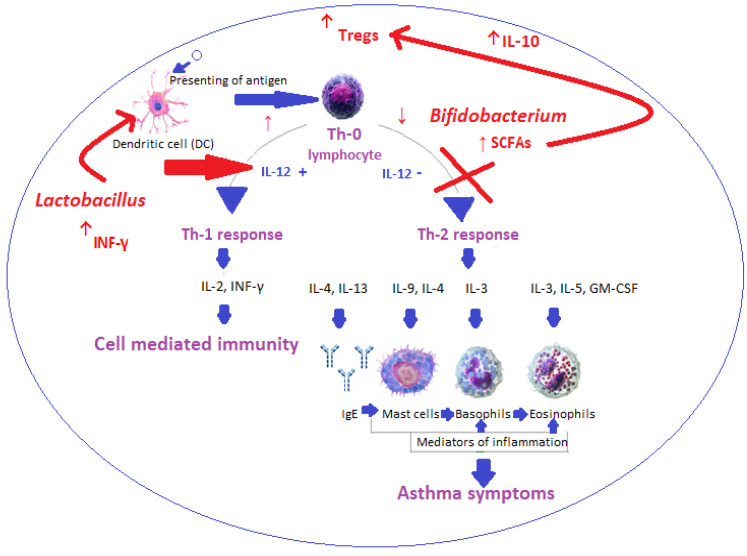
The mechanism of asthma development and the influence of probiotics on regulating Th1/Th2 balance (↑—increase; ↓ decrease/reduction; → influence on; thick green arrow—cause-and-effect chain).

In recent years, authors [19] have reported that the fermentation of dietary fiber by *Lactobacillaceae* and *Bifidobacteriaceae* in the intestine increases the level of SCFAs, leading to a reduction in inflammation associated with the Th2 response [20]. Moreover, butyrate, propionate, and acetate alleviate allergic airway inflammation *via* Tregs. *Lactobacillus* and *Bifidobacterium* increase the secretion of interleukin IL-10 [21] and inhibit the IgE-dependent immune response [22]. Probiotic microorganisms affect, among others, Th17 cells. Scientists [23] described that DCs cultured with *L. reuteri* and *L. casei* stimulate Th1 cells and Treg. Importantly, *Bifidobacterium* bacteria are more associated with the induction of anti-inflammatory and regulatory responses mediated by Treg [24], while *Lactobacillus* acts mainly by stimulating DCs to secrete IL-12 [25]. Probiotics have also been shown to increase the concentration of interferon gamma (INF-γ) [26] and support host immunity (Figure 2).

### 1.2. Asthma Affects the Bronchi

During asthma, the bronchial epithelium becomes more fragile [27]. The accumulating thickened mucus [27,28] is additionally penetrated by eosinophils, which have the ability to produce ROS. Importantly, the BALF of asthmatic mice contains an increased total number of exfoliated cells [29]. The exfoliation process is probably intensified by the activation of metalloproteinase by activated epithelial cells, resulting in disrupted cell–matrix connections. Loss of epithelial integrity may cause increased airways reactivity to stimuli through the release of cytokines and other proinflammatory mediators [30,31]. The extracellular matrix in the respiratory tract is altered, and the basement membrane begins to thicken early in the course of the disease. The above-mentioned factors result in the narrowing of the bronchial lumen and impeded airflow.

### 1.3. There Is No Effective Cure for Asthma

Treatment of the disease focuses on reducing the frequency of characteristic symptoms and alleviating their intensity. Generally, drugs used can be categorized as controlling, bronchodilating, or supportive. The first ones include medications taken chronically to permanently control the disease. These drugs reduce inflammation, control symptoms, and prevent exacerbations and worsening ventilation rates. Inhaled glucocorticosteroids [32], long-acting beta-2-agonists [33], and antileukotriene drugs [34] are used in this case. The second type are medications taken by the patient “on-demand” to provide relief. These drugs are intended to stop an attack of shortness of breath or prevent its occurrence, e.g., during physical exercise. Inhaled short-acting beta-2-agonists [33], anticholinergic drugs, and oral corticosteroids are used in this case. The last type of drugs describes monoclonal antibodies directed against human IgE immunoglobulin, IL-5, or the IL-4 and IL-5 receptors. These medications [6,35] are additionally administered in the case of severe asthma, or when symptoms persist despite the use of other drugs.

### 1.4. The Role of Microflora

**Figure 3:**
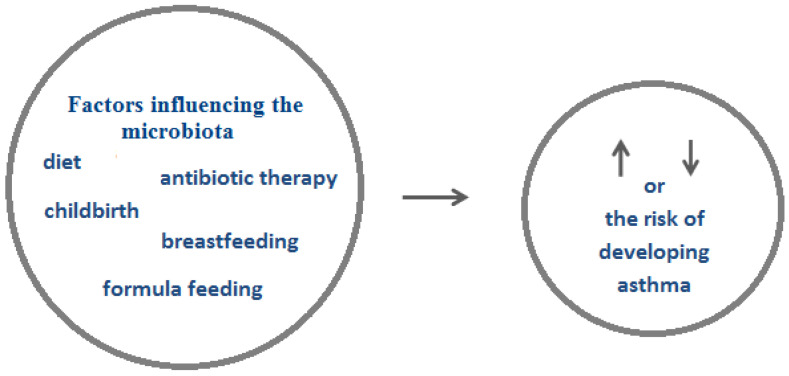
Selected factors influencing the microbiota (↑—increase; ↓—decrease).

Research points to the first three years of life as a key period for shaping the gut microbiome. During this time, dynamic changes in its composition and diversity occur, which are influenced by many factors. After this period, the microbiota stabilizes and its composition becomes similar to that present in adults. After birth, the intestine is rapidly colonized by a number of microorganisms, which is related to factors such as diet, or antibiotic therapy (Figure 3) [36].

The microorganisms that grow physiologically in the intestine not only reduce the development of pathogens, but also produce the ingredients necessary for proper functioning, such as short chain fatty acids (SCFAs) [37,38,39], which are produced in the process of carbohydrate fermentation. The presence of appropriate bacteria and the type of diet are the factors affecting this process. An important factor that affects the composition of the intestinal microflora in the first months of a child’s life is a diet consisting almost exclusively of milk, favoring fermenters such as *Bifidobacterium* [40,41,42]. Formula-fed babies have a significantly higher proportion of *Bacteroides* and the *C. coccoides* group [43]. A diet rich in plant-based carbohydrates increases the abundance of *Bacteroidetes*, which are specialized in their breakdown [44]. Interestingly, a lower abundance of these bacteria is correlated with obesity.

It is worth noting that the formation of a microflora is a gradual phenomenon. It is therefore possible to influence this development through an appropriate diet or/and probiotics [45,46,47,48]. A complete understanding of the gut–lung axis [49,50,51] may be crucial in the future management of asthma, although the mechanism by which these two systems may influence each other is not yet well understood. It seems likely that inflammation initiated in the intestines may result in inflammation in the lungs due to the destructive effects of an overstimulated immune system.

In recent years, scientists have been investigating how the microbiome itself can be modulated.

### 1.5. The Use of Probiotics and Prebiotics

According to the International Scientific Association for Probiotics and Prebiotics, the term “probiotics” cannot include non-living microorganisms or products derived from microorganisms [52]. The World Health Organization and the United Nations Food and Agriculture Organization define probiotics as live microorganisms that can pass through the digestive system alive and provide a benefit to the host [53] and “prebiotics” as non-digestible food ingredients that exert a beneficial effect on the host due to their ability to selectively stimulate the growth/activity of a specific number of bacteria in the intestines [54,55]. These compounds cannot be hydrolyzed or absorbed in the digestive tract. Their fermentation should induce a beneficial effect on the host’s system. The group of prebiotics includes dietary fibers, e.g., oligosaccharides. The most commonly used in asthma are inulin, fructooligosaccharides (FOS), and galactooligosaccharides (GOS) [56,57,58,59]. They are fermented by lactic acid bacteria, which generates the formation of SCFAs, e.g., butyrate which may have the potential to alleviate obesity and related comorbidities [60]. Another representative of SCFAs is propionate, which inhibits the initiation of the Th2 immune response by dendritic cells (DCs). Studies on animal models also confirm that this compound suppresses the M2 polarization pathway, thereby reducing allergic airway inflammation [61].

Probiotic microorganisms positively modulate the immune system by maintaining homeostasis between individual fractions of immune system cells. They can produce specific enzymes or metabolites that directly affect the microorganisms or influence the body by inducing its health-promoting effects. By producing antibacterial substances (bacteriocins, acids), competing for binding sites and nutrients, and modulating the immune system, probiotics directly block intestinal pathogenesis [62]. The most common strains used as probiotics in asthma are lactic acid bacteria—*Bifidobacterium* and *Lactobacillus*.

As the influence of microflora and bacteria on the incidence and development of asthma has become clearer, probiotics have become more popular as a “form of treatment/prevention”. However, their correct use requires more detailed knowledge of the patient’s microbiome.

### 1.6. Reactive Oxygen Species in Asthma

Reactive oxygen species (ROS) are produced as a result of natural cellular metabolic processes [63,64]. They usually have a positive effect on the body in moderate or low concentrations (they act in response to tissue damage, protect the body against pathogens via immune system cells, or participate in signal transmission in cells). However, their excessive production may lead to changes in cell components, modifying or inhibiting their functions, which leads to the development of many diseases, including atherosclerosis, diabetes, and asthma [65,66,67].

ROS are produced in oxidation processes that involve chemical reactions of electron transfer from one molecule, called the oxidant, to another, the compound, being oxidized. The result of such reactions may be both free radicals (FRs) and non-radical forms—neutral particles or ions [68]. FRs are formed as a result of free radical reactions: initiation, propagation (prolongation), and termination. FRs representatives include, among others, the most reactive hydroxyl radical, superoxide anion, or hydroperoxyl radical. The non-radical form is hydrogen peroxide [69]. It is a neutral, slightly reactive molecule that has the ability to penetrate cell membranes so it can be located in locations distant from its place of origin. The result of the reaction of this molecule with transition metals (mainly Fe^2+^ and Cu^+^) is the formation of a hydroxyl radical [70]. Exogenous sources of ROS are chemical compounds, such as drugs, pesticides, processed foods, alcohol, and physical factors, including industrial fumes, car exhaust fumes, cigarette smoke, ionizing radiation, ultrasound, ultraviolet radiation, and others. On the other hand, superoxide is produced mainly in the mitochondria [71].

**Figure 4:**
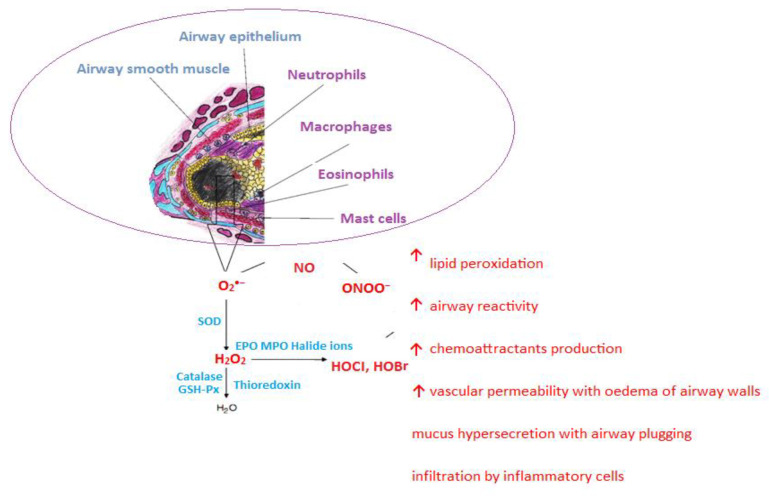
Oxidative stress in asthma—sources and pathophysiological effects (↑—increase; →↓—cause-and-effect chain, chemical reactions).

The overproduction of ROS, a deficiency of non-enzymatic antioxidants, or/and a reduction in the activity of the enzymatic antioxidant defense system causes a disturbance of the pro-oxidant–antioxidant balance—a phenomenon called oxidative stress (OS) [72,73,74]. OS participates in the pathogenesis of many diseases, including asthma (Figure 4) [75,76,77].

Chronic inflammation in asthma, with the participation of biological, chemical, or physical factors, leads to the development of OS. This leads to over-reactivity in the immune system and the activation of the production of inflammatory mediators. In patients with asthma, inflammation first dominates, and then oxidants interfere with the structure of goblet cells, which results in increased mucus production. Structural changes in the airways occur which are associated with bronchial remodeling. This in turn activates the secretion of inflammatory mediators and strengthens the disease state.

Moreover, based on the published studies, it can be concluded that there is a potential link between inflammation, oxidative stress, and dysbiosis in asthma.

### 1.7. Antioxidants Against ROS/OS

A proper cell activity requires maintaining a balance between the production of ROS and their elimination [78]. Antioxidants are substances that inhibit the oxidation of biomolecules and neutralize oxidants, transforming them into their non-reactive derivatives. Antioxidants form an extensive antioxidant defense system, which includes enzymes that decompose ROS and low-molecular-weight non-enzymatic compounds that transfer their electrons to FRs, thus undergoing oxidation [79].

Superoxide dismutase (SOD), catalase (CAT), glutathione peroxidase (GPx), and glutathione reductase (GR) are representatives of enzymatic antioxidants. Non-enzymatic antioxidants include compounds that occur naturally in the body (endogenous) and antioxidants that require dietary supplementation (exogenous). The second group includes compounds such as ascorbic acid (vitamin C), α-tocopherol (the form of vitamin E), vitamin A, flavonoids including anthocyanins, and sex hormones (estradiol, estrone) [80,81].

### 1.8. Antioxidants and Asthma

The factors contributing to the development of asthma, apart from tobacco smoke or air pollution (ROS generators), include an unhealthy diet. The modern diet is based on ready-made food products containing saturated fats, highly processed meats, refined carbohydrates, and various dyes, flavors, and preservatives. Lately, Luo et al. [82] demonstrated a causal association between the daily intake of sugars and fats, as well as the levels of magnesium and vitamin D in the serum, and the occurrence of childhood asthma.

Already, 30 years ago, scientists linked the increase in the prevalence and severity of asthma with deficiencies of antioxidants in food [83]. Scientists showed that a low vitamin E intake is associated with an increased incidence of asthma over a 10-year period [84]. Also, a study conducted in Saudi Arabia confirmed that low vitamin E levels are related to the occurrence of asthma [85]. Observational studies have found an association between a decrease in blood vitamin C concentration and an increased likelihood of asthma in children [86,87] and adults [88]. However, a study published in 2024 [89] does not confirm this association in adults. A 2023 meta-analysis [90] showed that serum vit. A levels are lower in asthmatics than in healthy controls. The effect of this vitamin may depend on age, stage of development, diet, and genetic conditions.

Some studies [91,92,93] suggest that an increased intake of antioxidants may lead to a reduced burden of severe asthma. In Larkin et al.’s [94] prospective study, α-tocopherol within normal reference ranges was associated with decreased asthma development. Tan et al. [95] demonstrated that vit. A-regulated ciliated cells repair the damaged airway epithelium caused by asthma and maintain the integrity of the airway epithelium. However, Checkley et al. [96] reported that early life vit. A administration in regions with chronic vitamin deficiency was not associated with a lower risk of asthma. A study published in the same year [97] on the use of flavonoids in the course of asthma confirmed a reduction in airway inflammation. Suna et al. [98] reported that asthmatic women exhibited lower total antioxidant status compared to the control group, but no significant differences were noted in the dietary intake of antioxidant micronutrients. The group of healthy participants had a significantly higher intake of anthocyanidins compared to asthmatics. The study suggests that a diet rich in flavonoids may help reduce inflammation and oxidative stress. Cho et al. [99] showed that treatment with soy isoflavones reduce the number of severe asthma exacerbations.

Interestingly, Murr et al. [100] observed that some foods rich in antioxidants and extracts of traditional Vietnamese and Chinese herbal medicines inhibit the secretion of the interferon-γ. This hypothesis, based on *in vitro* studies, argued that increased antioxidant intake by suppressing Th1 differentiation promotes Th2 differentiation due to inherent mechanisms of mutual regulation.

Thomas [101] suggested a significant association between deficiencies in micronutrients and the development of diseases. An article published in 2024 [102] showed a positive correlation between selenium intake and the lung function of asthmatics.

The results of an observational, controlled study [103] suggested that dietary supplements beneficially modulate plasma antioxidants and may therefore have a positive effect on systemic redox balance and, consequently, on pulmonary inflammation in asthmatic children.

**Figure 5:**
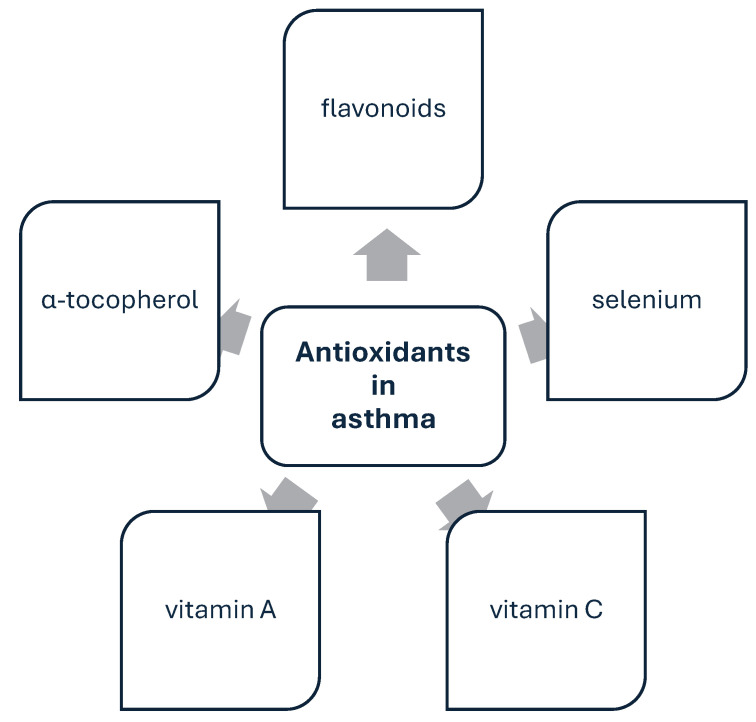
Antioxidants associated with asthma. The administration of some antioxidants, especially flavonoids, has a beneficial effect on the changes occurring in the course of the disease.

Antioxidants associated with asthma are shown in Figure 5.

### 1.9. Probiotics Against ROS/OS

Recent studies of probiotic strains have indicated their extensive involvement in defense mechanisms against ROS [104,105,106,107]. Some probiotics contain genes encoding antioxidant enzymes; thanks to this, selected strains have the ability to neutralize ROS directly in the gastrointestinal tract [108]. Probiotics have the ability to reduce inflammatory processes, which in turn protects the body against OS induced by proinflammatory cytokines [109,110,111,112]. The administration of probiotics into the digestive tract improves the bioavailability and absorption processes of micro- and macronutrients, including antioxidants. Moreover, they protect against the accumulation of ROS in the intestines during food intake, may reduce the production of ROS, and even reduce the phagocytic capacity of neutrophils (*L. rhamnosus*), one of the natural sources of ROS [109,111,112].

Scientific works have demonstrated the antioxidant properties of probiotics both *in vitro* and *in vivo* studies [113,114]. Lactic acid bacteria have a high intracellular concentration of total (GSHt) and reduced glutathione (GSH), and also show higher TAA compared to the control (usually *E. coli* bacteria) [115,116]. The use of *Enterococcus faecim* with FOS resulted in a reduction in the level of GSHt in the blood of birds [117]. Most of the experiments conducted so far have shown that the activity of probiotics is associated with an increase in the concentration of GSHt in the tissues [106,118,119,120]. Erginel et al. [121] observed a beneficial effect of probiotics on GSHt concentration. In another study [118], scientists showed that the administration of *L. rhamonus* significantly increases GSHt concentration. Similarly, in another mouse model [122], administration of *L. rhamonus* bacteria resulted in an increase in the concentration of GSHt. Verma and Shukla observed that probiotics and synbiotics cause an increase in tissue GSH concentration in rats [111]. Similarly, in animals treated with doxorubicin, a protective effect of a mixture of probiotic bacteria was observed on the plasma concentration of GSH. Lutgendorff et al. [114] observed an increase in GSH concentration in the rat plasma, probably caused by the intensification of its *de novo* synthesis. Sengül et al. [123] described the beneficial effect of probiotic supplementation on reducing the concentration of GSSG. In 2022, the authors presented data confirming that probiotic preparation enhances antioxidant activity and leads to an increase in serum SCFA concentration in mice [124].

Human trials were conducted by Asemi et al. [119,125], Bahmani et al. [126] and Taghizadeh et al. [127]. Importantly, the use of a preparation composed of many probiotic genera resulted in a significant increase in the concentration of GSHt in the blood plasma [119]. The combination of *L. sporenges* with inulin had a similar effect [123]. A combination of probiotics and prebiotics was used in another study [126,127]. Another experiment [128] confirmed the ability of probiotics to increase the concentration of GSHt in plasma. Cannarella et al. [129] reported that a bacterial preparation reduces inflammatory biomarkers and improves the oxidative–nitrosative profile. The results of the randomized controlled trial [130] indicated that *Saccharomyces boulardii* significantly improves TAC and slightly reduces malondialdehyde (MDA) levels, thereby reducing OS. Similar results were obtained by Farajipour et al. [131]. The authors of the next study [132] showed that the administration of the probiotic significantly reduced the levels of MDA, LDL cholesterol, and tumor necrosis factor-α. Juan et al. [133] described that probiotic supplementation significantly improved cognitive impairment. The authors of the next study [134] reported that probiotic preparation caused a significant increase in TAC levels.

### 1.10. Probiotics and Asthma

**Figure 6:**
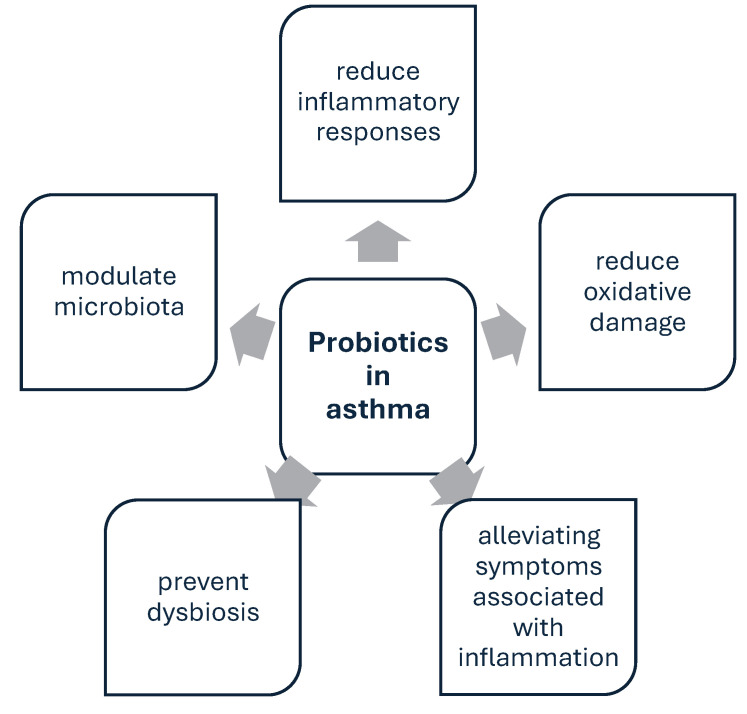
Probiotics functions in the airway. The administration of probiotics has a beneficial effect on the changes occurring in the course of the disease.

The number of scientific reports regarding the use of probiotics in asthma has increased significantly in recent years. The functions of probiotics in the airway are presented in Figure 6.

**Table 1:** The use of probiotics in scientific research in the last 5 years in relation to asthma.

	Data	Objective/Model/Probiotics	Results	References
Type of Study				
*In vitro*		construction of the pBESIL10 vector by cloning the human IL-10 gene under a *gap* promoter and signal peptide from *Bifidobacterium* spp. into the *E. coli*-*Bifidobacterium* shuttle vector pBES2; functional evaluation of the cell-free culture supernatant of *B. bifidum* BGN4 [pBESIL10]	efficient production and secretion of significant amounts of biologically active human IL-10; reduction in IL-6 production in LPS-induced Raw 264.7 cells and IL-8 production in LPS-induced HT-29 cells or TNFα-induced HT-29 cells	Hong et al. 2021 [135]
		metabolic footprint of cell cultures of 25 commercially available probiotic strains (metabolic pathway activities with their corresponding immunomodulatory activity)	an overrepresentation of the tryptophan metabolic pathway for the bioactive supernatant class—molecules involved in this pathway may be involved in immunomodulatory activity	Fonseca et al. 2022 [136]
*In vivo* (animals)		HDM-induced allergic inflammation; mice; *i.r.* inoculation of the active component-overexpressing Clear *coli* + *i.p.* injection of recombinant component protein	a novel mechanism of moonlighting LGp40 in the reversal of M2-prompted Th2 cell activation through glycolytic activity (immunoregulatory role in the prevention of allergic asthma)	Chen et al. 2022 [137]
		OVA-induced allergic inflammation; mice CCFM1228, FBJSY202, FHNXY26M4, FNMGHHHT2M2, CCFM1274, SHXXA4M1T78, ZJHZD20M6, and CJ653	*B. animalis* subsp. lactis CCFM1274, SHXXA4M1T78—reduction in serum OVA-sIgE levels and peribronchial, perivascular cellular infiltration, and IL-17, IL-10 production in BALF	Wang et al. 2024 [138]
		HDM-induced allergic inflammation; mice heat-killed *A. muciniphila* EB-AMDK19 (AMDK19-HK)	suppression of Th2-dependent immune responses; protective effect against the development of asthma	Yoon et al. 2024 [139]
		OVA-induced allergic inflammation; mice *L. plantarum* APsulloc331261 (GTB1TM)	alleviation of allergic airway inflammation and reduction in excessive mucin secretion *via* butyrate production	Kim et al. 2024 [140]
		OVA-induced allergic inflammation; rats *L. paracasei* 33 (LP33)	reduction in the total number of inflammatory cells, lymphocytes, and eosinophils (BALF) decreased in the level of IgE and cytokines in Th2	Yang et al. 2022 [141]
		BP aeroallergen-induced allergic inflammation; mice *L. rhamnosus* GG, GR-1	reduction in eosinophils count (BALF), IL-13 and IL-5 (lungs) and AHR—LGG only	Spacowa et al. 2019 [142]
		OVA-induced allergic inflammation; mice *L. rhamnosus* 76 (LR76)	reduction in IL-4, IL-5, IL-13, and IL-25 levels; inhibition of mucus secretion in respiratory epithelial cells by reducing the expression of the STAT6/SPDEF pathway	Hou et al. 2023 [143]
		Der p 2-induced allergic inflammation; mice *L. rhamnosus* GG+/or prednisolone	reduction in airway resistance and serum IgE, IgG1, and IL-4, IL-5, IL-6, IL-8, IL-13, and IL-17, and increase in serum IgG2a	Voo et al. 2022 [144]
		Bet v 1-induced allergic inflammation; mice *L. rhamnosus* GR-1	preventing the deterioration of respiratory function and promoting the immunity of the intestinal microbiome	Spacova et al. 2020 [145]
		HDM-induced allergic inflammation; mice *L. rhamnosus* GG+/or turmeric	amelioration of AHR; reduction in eosinophilia, IL-5, IL-13, and CCL17 (only with prebiotic)	Ghiamati et al. 2020 [146]
		OVA-induced allergic inflammation; mice *L. paracasei* K47	amelioration of AHR and inflammation; reduction in total serum IgE, OVA-specific IgE and OVA-specific IgG1; regulation of Th1/Th2 balance	Chen et al. 2022 [147]
		OVA-induced allergic inflammation; mice *L. plantarum* CQPC11	reduction in TNF-α, IL-4, IL-5, IL-6, and 13 (BALF); reduction in histological edema, IgE, OVA-specific IgE, and IgG1	Lan et al. 2022 [148]
		OVA-induced allergic inflammation; mice *L. delbrueckii* UFV-H2b20	reduction in IgE, eosinophils, monocytes, and alveolar macrophages; increased cytokine ratio IFN-γ/IL-4, increased pulmonary IL-10, CD39 + CD73+ regulatory T cells	Montuori-Andrade et al. 2022 [149]
		HDM-induced allergic inflammation; mice *L. casei*	increase in acetate and propionate content depending on strain; increase in sIgA and IL-10	Li et al. 2020 [150]
		OVA-induced allergic inflammation; mice *L. plantarum* RGU (Lp-1)	increase in expression of IL-10, decrease in expression of IL-1β, IL-13, and IL-17 in lymphoid tissue	Kishida et al. 2022 [151]
		OVA-induced allergic inflammation; mice *L. bulgaricus* N45.10	increase in T-bet, Foxp3; attenuation of inflammation and airway remodeling	Anatriello et al. 2019 [152]
		OVA, DEPM-induced allergic inflammation; mice *L. plantarum* GCWB 1001, *L. rhamnosus* GCWB1156, *Pediococcus acidilactici* GCWB1085	reduction in induced inflammatory infiltrate, goblet cell hyperplasia, airway remodeling, proinflammatory cytokine, and chemokine levels in BALF	Jin et al. 2020 [153]
		OVA-induced allergic inflammation; mice *B. infantis*	AHR reduction, Th2-related cytokine reduction BALF/lung IL-4, IL-5, IL-13; increase in Th1-related cytokines-increase BALF/lung IFN-γ; reduction in eosinophil, neutrophil, and macrophage content in BALF	Wang et al. 2020 [154]
		HDM-induced allergic inflammation; mice *L. reuteri*, *L. rhamnosus*, *L. fermentum*, *L. salivarius*, *L. gasseri*, *L. casei*	increased butyrate production, alleviating airway inflammation, and Th2 response in lung tissue	Li et al. 2020 [155]
		OVA-induced allergic inflammation; mice *S. cerevisiae* UFMG A-905	reduction in AHR and lung inflammation in a dose-dependent manner	Milani et al. 2024 [156]
		OVA-LPS-induced allergic inflammation; mice *L. acidophilus* LA-5+ *L. rhamnosus* GG+ *B. animalis* subspecies lactis BB-12	reduction in AHR, BALF eosinophils, IL-17, EPO activity	Wu et al. 2022 [157]
		OVA-induced allergic inflammation; mice *L. gasseri* LK001 + *L. salivarius* LK002 + *L. johnsonii* LK003 + *L. paracasei* LK004 + *L. reuteri* LK005 + *B. animals* LK011	immunomodulatory effects (accumulation of gut-primed Foxp3 + Treg induced by MLN CD103 + DC, which can migrate to the lung through the lymph and/or bloodstream)	Zhang et al. 2021 [158]
		OVA-induced allergic inflammation; mice *B. breve* Bif11+ *L. plantarum* LAB3	acetic acid and butyric acid levels returned to normal to a moderate but significant degree	Monga et al. 2023 [159]
		HDM-induced allergic inflammation; mice *B. tetravaccine*+a mixture of bacterial lysates	increase in proportion of Tregs in peripheral blood; reduced risk of asthma only in offspring of mothers with a high microbiological load	Li et al. 2020 [160]
*In vivo* (human trails)		children and adolescents *L. reuteri* DS 17938	improved values of C-ACT, increased PEF, and reduced the number of symptoms	Moura et al. 2019 [161]
		children *L. salivarius* LS01 (DSM 22775) + *B. breve* B632 (DSM 24706)	reduced the frequency of asthma exacerbations	Drago et al. 2022 [162]
		children *L. casei*, *L. acidophilus*, *L. rhamnosus*, *L. bulgaricus*, *B. infantis*, *B. breve*, *S. thermophiles* + FOS	reduction in the number of outpatient hospital visits due to asthma-related problems (no probiotic-only group)	Hassanzad et al. 2019 [163]
		*L. casei*, *L. acidophilus*, *L. rhamnosus*, *L. bulgaricus*, *B. breve*, *B. longum*, *S. thermophilus* + FOS	improvement of FEV and FVC values (no probiotic-only group)	Abbasi-Dokht et al. 2023 [164]
		adults *L. acidophilus* LA-5, *L. rhamnosus* GG, *B. animalis* subspecies lactis BB-12+/or inulin	improvement of airway inflammation, asthma control, and gut microbiome composition	McLoughlin et al. 2019 [165]
		adults Probio-M8 powder + Symbicort Turbuhaler	reduction in asthma symptoms, reduction in exhaled nitric oxide fraction, and improvement of asthma control test results	Liu et al. 2021 [45]
		adults *L. reuteri* DSM-17938	no evidence that DSM-17938 exerts any systemic effects on airway nerves, smooth muscle, sputum inflammatory cells, skin reactions, or T cell responses	Satia et al. 2021 [166]
		adults *L. casei*, *L. acidophilus*, *L. rhamnosus*, *L. bulgaricus*, *B. breve*, *B. longum*, *S. thermophilus* +FOS	effects on IL-6, IL-17, and TGF-β associated with Th17 cells; and on FEV1 and FVC values (neutrophilic asthma)	Sadrifar et al. 2023 [167]
		children *L. rhamnosus* GG	a *bifidobacteria*-dominant gut microbiome is more often associated with LGG supplementation and better clinical outcomes	Ray et al. 2022 [168]

### 1.11. Lactobacillus and Bifidobacterium Are Still the Best-Studied Bacteria

In animal studies, apart from the above-mentioned bacteria, the influence of *Enterococcus faecalis* and *Saccharomyces cerevisiae* UFMG A-905 on the course of this disease was also analyzed.

*Lactobacillus rhamnosus* bacteria have been studied quite intensively in animal model studies. The LR76 strain reduced inflammation and mucus secretion in airway epithelial cells by reducing the expression of the STAT6/SPDEF pathway [143]. The authors [144] reported that the combination of LGG with prednisolone reduces concentrations of IL-4, IL-5, IL-6, IL-8, IL-13, IL-17, increases serum IgG2a concentration, and also decreases airway resistance and IgE and IgG 1 concentrations. Administration of the LGG strain in other studies [142,146] led to a reduction in airway hyperresponsiveness (AHR) and eosinophilia, as well as a decrease in the concentrations of IL-5 and IL-13. The wild-type and recombinant *L. rhamnosus* GR-1 prevented respiratory function deterioration and supported gut microbiome immunity [145]. *L. plantarum* RGU (Lp-1) increased IL-10 expression and decreased the concentration of IL-1β, IL-13, and IL-17 in the lymphoid tissue, while the CQPC11 strain reduced the concentration of TNF-α, IL-4, IL-5, IL-6, and IL-13 in the bronchoalveolar lavage fluid (BALF), histological edema, and ovalbumin (OVA)-specific IgE, IgE, and IgG1 [148,151]. An experiment [141] conducted on SD rats proved that *L. paracasei* 33 reduces the total number of inflammatory cells, lymphocytes, and eosinophils in the BALF and the level of IgE and cytokines in Th2 cells. However, *L. delbrueckii* UFV-H2b20 reduced lung IgE, eosinophils, monocytes, alveolar macrophages, and increased the IFN-γ/IL-4 cytokine ratio, lung IL-10, and CD39 + CD73+ regulatory T cells in a mouse study [149]. Scientists reported that *L. bulgaricus* N45.10 increases anti-inflammatory cytokines and inhibits inflammation and airway remodeling by interfering with the Th1/Th2 cytokines and STAT6/T-bet transcription factors [152]. *L. casei* bacteria increased acetate and propionate content depending on the strain [150]. *Saccharomyces cerevisiae* UFMG A-905 has been shown to reduce AHR and lung inflammation in a dose-dependent manner [156]. In contrast, *Enterococcus faecalis* showed no protection against allergic asthma in mice but increased the concentration of SCFAs in offspring [169]. The authors described that *B. infantis* promotes Th1 immune response and inhibits Th2, and the CGMCC313-2 strain reduces allergic inflammation in mice [154,170]. Probiotic preparations composed of mixed strains or in combination with prebiotics significantly reduced AHR in mice [157,159]. The use of *B. tetravaccine* and a mixture of bacterial lysates led to an increase in Tregs in peripheral blood, but only in the offspring of mothers with a high microbiological burden was the risk of disease decreased [160].

The scientists examined various genus, species, and strains of probiotic bacteria in human trials such as the following: *Lactobacillus* (*rhamnosus* GG HN001, *acidophilus* LA-5, *salivarius* LS01 DSM 22775, *reuteri* DSM-17938, *bulgaricus* N45.10, *casei* DN001, *paracasei* or *fermentum*), *Bifidobacterium* (*lactis* 420 DSM 22089, BB12, *breve* M-16 V, B632 DSM 24706, *ifantis* M-63, 35624, *longum* BB536), and *Streptococcus thermophilus*—only in the mixture.

Studies on the use of probiotics in preventing this disease in humans are inconclusive. Recent reports do not confirm this thesis [171,172,173], although in accordance with World Allergy Organization (WHO) recommendations, probiotics should be taken by pregnant women who are at high risk of having an allergic infant [174].

The administration of *L. reuteri* DS 17938 as an adjunctive therapy in the treatment of children and adolescents with asthma led to improved values of Childhood Asthma Control Test (C-ACT), increased peak expiratory flow rate (PEF), and a reduced number of symptoms [161]. Similarly, the combination of *L. salivarius* LS01 (DSM 22775) and *B. breve* B632 (DSM 24706) significantly reduced the frequency of asthma exacerbations in children [162]. Also, the combination of *L. paracasei* with *L. fermentum* resulted in the improvement of PEF, the reduction in IgE concentration in blood, and the improvement of the C-ACT values [175].

**Figure 7:**
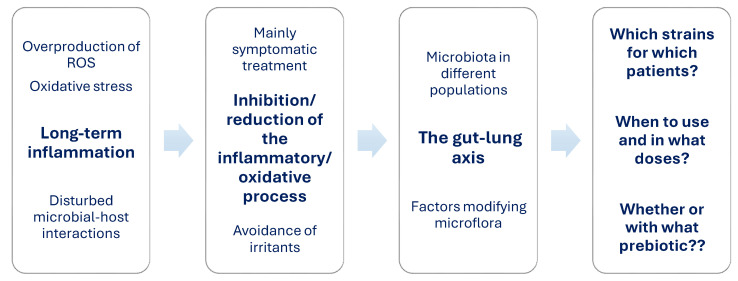
Conclusions from the use of probiotics/prebiotics in asthma therapy.

The use of multi-strain probiotics with prebiotics has proven to be very effective. The combination of *L. casei*, *L. acidophilus*, *L. rhamnosus*, *L. bulgaricus*, *B. infantis*, *B. breve*, *S. thermophiles,* and fructooligosaccharide (FOS) in the treatment of children with asthma significantly reduces the number of outpatient visits to the hospital due to asthma-related problems [163]. The administration of a mixture consisting of *L. casei*, *L. acidophilus*, *L. rhamnosus*, *L. bulgaricus*, *B. breve*, *B. longum*, *S. thermophilus* and FOS improves forced expiratory volume (FEV) and forced vital capacity (FVC) [164]. The researchers also found improvements in airway inflammation, asthma control, and gut microbiome composition after inulin administration. In the experiment, asthmatics took *L. acidophilus* LA-5, *L. rhamnosus* GG, *B. animalis* subspecies lactis BB-12, and/or inulin [165]. A recent study [45] has proven that the combination of probiotic microorganisms with budesonide alleviates asthma symptoms, reduces the fractional exhaled nitric oxide level, and improves the asthma control test score. Table 1 shows scientific studies on the use of probiotics in asthma from the last 5 years, while Figure 7 presents conclusions regarding the role of probiotics in asthma therapy.

### 1.12. Prebiotics and Asthma

In recent years, prebiotics have been added as a complementary agent to stimulate probiotic activity (especially in human trials). Prebiotic fermentation can modulate the composition and function of probiotic microorganisms. Prebiotics with regulatory abilities modify the microbiota to a favorable state. It should be emphasized that the combination of one or more prebiotics with a probiotic is more effective than when they are used separately [165,176,177]. Prebiotics have less influence on the control of eosinophilic airway inflammation, EPO activity, immune-allergic response, and asthma. The authors confirm that short- and long-chain prebiotics (GOS and FOS), together with probiotics, prevent allergic sensitization by regulating immune responses. Probiotic microorganisms can modulate immune cells such as T1, T2, T17, Treg, and B cells [178,179]. Some studies have shown that prebiotic supplementation improves airway hyperresponsiveness and reduces the number of inflammatory cells in the sputum of asthmatics [165,180,181]. Inulin (12 g/day) has also been shown to improve airway inflammation, asthma control, and gut microbiome composition [163]. Additionally, Wu et al. 2022 [157] showed that prebiotic treatment (GOS and FOS; 10 mg/kg, b.w.) significantly inhibited PI3K expression.

Prebiotic fiber, by the production of acetate, prepares Treg cells to protect against asthma. Prebiotics, such as soluble fiber and inulin, must be fermented by beneficial bacteria, which is a time-consuming process. Therefore, these products cannot act quickly and have anti-inflammatory properties [165,182].

## 2. Conclusions

The administration of probiotic microorganisms positively modulates the immune system by, *inter alia*, maintaining homeostasis between individual fractions of immune system cells. Although these organisms have a clear influence on the processes occurring in asthma, the complex mechanisms of action and bacteria–host interactions remain poorly understood. *In vitro* studies have allowed for a more precise understanding of the mechanisms of probiotic action and their impact on asthma, and these data have been supplemented and expanded by animal experiments and clinical studies, the number of which is still insufficient.

The administration of probiotics, prebiotics, and/or antioxidants has a beneficial effect on the course of the disease. Studies suggest that a diet rich in flavonoids may help reduce inflammation and oxidative stress. In recent years, scientists have described that probiotics reduce AHR or mucus secretion in the airway, and multi-strain preparations show promising results in the treatment of clinical symptoms of asthma, improve parameters of forced expiratory volume, peak expiratory flow, and reduced inflammation. However, our current understanding of the individual response to probiotic therapy, and the effects of its combination or doses, remains insufficient.

## Abbreviations

AHR	airway hyperresponsiveness
BALF	bronchoalveolar lavage fluid
CAT	catalase
FOS	fructo-oligosaccharide
FRs	free radicals
GOS	galacto-oligosaccharide
GPx	glutathione peroxidase
IL	interleukin
OS	oxidative stress
OVA	ovalbumin
PEF	peak expiratory flow rate
ROS	reactive oxygen species
SCFAs	short-chain fatty acids
SOD	superoxide dismutase
